# Doing more with less: the impact of the COVID-19 pandemic on Latino-serving community-based organizations

**DOI:** 10.1186/s12889-025-25490-2

**Published:** 2025-12-16

**Authors:** Maria-Elena De Trindad Young, Brenda Castañeda-Castañeda, Damian Juarez-Cahue, Kesia Garibay, Gabriel Chamie, Carina Marquez, Kara Marson, Irene H. Yen

**Affiliations:** 1 Public Health Department, School of Social Sciences, Humanities and Arts, University of California, Merced, 5200 N Lake Road, Merced, CA 95343 USA; 2 Division of HIV, Infectious Diseases and Global Medicine, San Francisco General Hospital, University of California, San Francisco, 2540 23rd Street, San Francisco, CA 94110 USA

**Keywords:** Community-based organizations, Latino, COVID-19 pandemic, Funding, Community-academic partnership

## Abstract

Community-based organizations (CBOs) played a key role in providing health and safety net support to low-income Latino communities during the COVID-19 pandemic; yet we know little about how organizations navigated challenges during this time. Understanding how CBOs navigate challenging periods is critical to ensuring a robust safety net for Latino and other underserved populations. We examined the factors that allowed CBOs to address Latino communities’ needs and the potential long-term impact of the pandemic on the sustainability of their health programs. Using a case study approach, we conducted in-depth interviews with directors, program managers, frontline staff, and community health workers from three CBOs serving Latinos in California. Interviews were analyzed in their original language (English or Spanish) to identify three key themes. Bilingual, bicultural CBO staff were key resources in providing tailored interventions to Latino communities. The pandemic led to an infusion of new funding that allowed CBOs to expand staff capacity and engage in health promotion initiatives for the first time. However, despite the new infusion of resources, the ongoing lack of flexibility from funders, challenges to providing pay to community health workers, and financial burdens of grants left CBOs in precarious circumstances. This examination of how CBOs adapted during the COVID-19 pandemic provides insights into how organizations navigate crises and other major changes in health needs and the funding landscape and identifies the resources needed to maintain the sustainability of the organizations that provide critical safety net services to Latino and other underserved populations.

## Introduction

Due to the COVID-19 pandemic, between 2020 and 2023, the Latino population in the U.S. experienced disproportionately high rates of COVID-19-related morbidity and mortality [1–3]. During this period, community-based organizations (CBOs) played a key role in providing health, economic, and social safety net support to low-income, immigrant, and Latino communities [4]. CBOs served as the trusted partner for both governmental and academic initiatives aimed at preventing the spread of COVID-19 while adapting their services, priorities, and financial stability under crisis circumstances [5]. We know little about how Latino-serving CBOs’ navigated the pandemic. This study fills that gap by examining the factors that allowed CBOs to address Latino communities’ needs and the pandemic’s potential long-term impact on organizational sustainability.

In California, about 1 in 10 of the 44,569 registered 501(c)3 nonprofit organizations provide health care services [6]. Nearly 500 nonprofits expressly address issues related to Latino populations [7]. CBOs work directly with community members and often include members of the underserved population in their efforts, such as through Community Health Worker (CHW) or *promotores/as* programs, to disseminate linguistically accessible and culturally tailored health interventions [8–10]. CBOs generally work with limited resources. Funding is largely tied to short-term projects with specific activities and extensive reporting requirements [6]. Most fund their services through an “interdependent model” relying on government grants, contracts, and maintaining donor networks and agencies [11, 12]. 

During the COVID-19 pandemic, CBOs serving communities of color were recognized for being in a unique position to rapidly leverage and expand existing community trust to respond to local needs [13]. Local, state, and federal governments have historically relied on CBOs to quickly administer disaster services and relief [14]. Numerous governmental pandemic funding initiatives, such as the Coronavirus Aid, Relief, and Economic Security (CARES) Act, brought health and financial resources to communities; and National Institutes of Health (NIH) initiatives, such as the Rapid Acceleration of Diagnostics-Underserved Populations (RADx-UP) program, supported community-engaged efforts [15, 16]. With pandemic funding, Latino-serving CBOs contributed to health efforts, from distributing nutrition, financial, and other resources to providing access to COVID-19 testing and addressing vaccine hesitancy.

Recent studies have evaluated the impact of interventions led by or in partnership with CBOs [5, 10, 17]. These indicate that CBOs are well-positioned to leverage existing trust with communities, use tailored approaches, and respond to sociopolitical, geographic, and cultural factors during health crises like the COVID-19 pandemic [5]. CBOs used various community-engaged models, such as training CHWs to be trusted community leaders who could address vaccine hesitancy; [10] conducting mass campaigns, such as “Get Out the Vaccine” efforts; [5] and adapting strategies for specific race/ethnic populations [17]. 

This research, however, has focused on CBOs’ effectiveness rather than the organizations themselves. Little is known about the impact of the pandemic on Latino-serving CBOs’ financial stability, organizational practices, or staff capacity. Yet, one survey from 2020 estimated that, nationally, 30% of nonprofits experienced financial challenges [18]; and the few studies that have documented the early impact of the pandemic on CBOs showed that most experienced disruption of services, changes in staff roles, challenges in delivering services while keeping staff safe, and technology barriers [11, 19–22]. 

Over five years since the official declaration of a global pandemic, it is critical to understand the organizational experiences of Latino-serving CBOs to identify their needs in future health crises and to ensure that they emerged from the pandemic with the support to continue their work in Latino communities. We sought to understand how Latino-serving CBOs sustained themselves as they responded to the pandemic, the factors that enabled them to respond to community needs, and how these experiences shaped their future priorities and sustainability.

## Methods

We conducted a case study of the organizations that participated in an NIH RADx-UP-funded project in California. We conducted and analyzed in-depth interviews with staff in a range of direct service and leadership roles. The study received approval from the University of California, San Francisco Institutional Review Board.

### Sampling and recruitment

We leveraged an existing community-academic partnership, the Latino COVID-19 Collaborative (LCC), located in California, funded through an NIH RADx-UP grant. The LCC aimed to increase access to COVID-19 testing by sharing knowledge and strategies between an academic partner and three Latino-serving CBOs in two Northern California communities. Prior to the pandemic, the CBOs had focused on services such as poverty alleviation, community development, and health promotion. While each relied on grants for their work, they entered the pandemic with distinct funding compositions: one with well-established local government grants; another that served primarily as a fiscal pass-through for smaller nonprofits; and the third a newly formed organization reliant on small, project-focused grants. Through the partnership, the three CBOs met monthly with the academic partner who provided updated information and capacity development regarding COVID-19 information [4] and facilitated the collaborative development and implementation of testing strategies, including mass testing events in communities and ongoing testing outreach [23]. The three CBOs in this partnership offered an ideal sample as they shared at least one funding source while also representing a diversity of organizational focuses, sizes, and funding models among Latino-serving CBOs.

To recruit participants, we provided the CBOs with a description of the study and asked them to provide a list of staff willing to participate in an interview and sent email invitations to each. Our interview guides, tailored to leadership and direct services staff, asked about the organizational impacts of the pandemic, services, partnerships, finances, staff capacity, and professional challenges unique to serving Latino populations.

### Data collection and analysis

We assigned the CBOs the following pseudonyms: Generations Rising, Community Hub, and Healthy Horizons. Participants provided signed informed consent and created a pseudonym. We interviewed 16 participants. Based on their description of their role, we categorized participants as executive directors (*n* = 2), program managers (3), program assistants (7), or CHWs/*promotoras* (4). From April-June 2023, interviews, ranging from 51 to 104 min, were conducted (by Authors 1, 2, and 4) and recorded through Zoom in English or Spanish and were then transcribed in their original language. Participants received a $25 e-gift card.

### Analysis

Deidentified interview transcripts were coded in their original language in Dedoose. We developed our codebook through a deductive approach [24]. Based on themes in the literature, we generated an initial set of codes related to the topics: COVID-19 impact and response; financial challenges; staff capacity; organizational structure; future sustainability; and Latino community needs. We tested the codes on three transcripts, adding additional codes. Three members of the research team coded the transcripts; the principal investigator (Author 1) reviewed a selection of coded transcripts and the team met regularly to discuss challenging excerpts to ensure reliability. The research team reviewed the content of the coded excerpts to derive three themes, discussed below, that describe factors that enabled CBOs to support their communities during the pandemic and the pandemic’s impact on their priorities and sustainability.

## Results

Each CBO provided services through distinct models. Generations Rising, the largest of the three, had long-established funding for poverty alleviation programs like nutrition resources (e.g., a food bank), youth development programs, and job training. Community Hub, while not explicitly focused on Latino populations, operated in a predominantly Latino community and geared services towards Latino residents. They focused on strengthening the local “CBO ecosystem” by advancing partnerships among smaller CBOs, including Healthy Horizons, the third CBO, which coordinated a CHW/*promotora* network to build community capacity. Prior to the pandemic, CBO leaders reported facing challenges with funders, county health departments, academics, and others, who had pre-determined assumptions on resource allocation, requiring CBOs to advocate for their inclusion in decision making.

### Bi-cultural and mission-driven staff were a critical resource

CBO staff were committed to serving their communities and achieving their organizations’ missions – before and during the pandemic. Staff were critical resources because they were bi-cultural and could gain the trust of the community. These staff included both CHWs/*promotoras* and program assistants, both of whom described their strong relationships with community members. As members of Latino communities, staff understood the needs and barriers in their community. This understanding was particularly critical during the pandemic. A CHW/*promotora* commented: “They were smart to call in people from the community because it’s not the same if someone comes in with ideas who doesn’t live here …to have brought in all of these women from different [Latin American] countries made it easier to understand the community. That was a point in our favor.”

CHW/*promotoras* and program assistants engaged the community where they were, including organizing community health events and bringing testing and vaccine resources to flea markets, Latino supermarkets, and Latino-run businesses. CHW/*promotoras* used their personal experience and knowledge of the community to build trust and disseminate information regarding COVID-19 in Spanish in an easy-to-understand manner. As a result, they observed improved access in their communities, such as increased COVID-19 testing and vaccinations.

While staff were a critical resource, CBOs needed funding to dedicate staff time to pandemic-related efforts. As we describe below, they were ultimately able to connect with partners and obtain funding to advance COVID-19 relief efforts, but such partnerships and funding connections did not exist prior to the pandemic, and energy went into finding these opportunities. CBO leaders reported that in the early months of 2020, they sought but struggled to find funding for COVID-19 relief efforts. As one program manager described it: “there was a lot of work that we did, a lot of advocacy as far as trying to find resources.”

### Pandemic funding expanded staff and capacity to engage in health interventions

Ultimately, the CBOs were invited to the RADx-UP partnership and received funding to address the pandemic. This new funding infused the resources to invest in current and new staff. The CBOs hired more staff and provided them with professional development. Generations Rising created and Healthy Horizons expanded programs for training community members to deliver health interventions. As many CBO staff shifted to remote work, local residents came forward to volunteer; in recognition of their efforts, these community leaders were offered official positions and were compensated for their work in the community. A CHW/*promotora* described how she was recruited: “We donated masks and that’s how they got my phone number…Later, they called me to see if I wanted to come volunteer.” A director observed: “We always knew the importance of *promotoras*, but COVID really proved the difference having *promotoras* on your team to help local communities, build more partnerships and really connect the community and get more people engaged.”

In addition to CHWs/*promotoras*, newly hired staff included bilingual, bicultural college students and recent college graduates interested in pursuing public health careers. The collaboration between them and the CHWs/*promotoras* was uniquely effective as the young professionals had the same bilingual and bicultural skill sets as CHWs/*promotoras* and possessed skills to bridge a technological gap between the Latino community and CBOs leadership. Two of Generations Rising’s program assistants got involved with the CBO when their mothers started working as CHWs/*promotoras*: “Of these volunteers that were helping, there were mom and daughter teams. We ended up hiring [the daughters] as our health specialists. Let’s nab them! They are bilingual. They have the technological skills that we require. So, they are going to be the ones that bridge the technology gap with the community,” explained a program manager. One of the program assistants described their experience advancing within the CBO: “We started off as interns. But as we progressed in our work here and saw that the community had more needs, we started working full-time, and that’s when we transitioned to a health specialist. That’s when our title changed, and we were given a couple more roles in our job as well.”

A second key outcome of the infusion of pandemic funding was that CBOs were able to work on health issues for the first time. Neither Generations Rising nor Community Hub had previously worked on health-focused projects. One director was involved in a very different area of focus: “…at the [beginning of the pandemic] I was part of a different organization that was under Community Hub…that does a lot of things on education and political education.” However, the directors all reported that the pandemic had thrust community health into the forefront of their priorities: “We couldn’t focus on education or workforce development or entrepreneurship unless we responded to what was in front of us, right?” The pandemic resulted in a context in which CBOs not only became involved in health issues but recognized their ability to expand their work to promote health beyond COVID-19 issues.

At the time of the interviews, as pandemic interventions were winding down, Generations Rising was working with the community to develop mental health support groups and register people for their county’s disaster text message notification service. They attributed participation in COVID-19 projects to the impetus to create a “health department” within the organization; a program manager noted “Something that we are grateful for with this [RADx-UP-funded project] is that it has helped us create this health department.”

Community Hub was continuing to focus on pandemic-related relief, including leading a new farmworker financial relief program in their county. In early 2023, catastrophic floods struck one of the farmworker communities they worked with. They recognized new community priorities and, with approval and support of the funder, shifted staff time from testing and other pandemic-focused outreach, to distributing emergency food kits, assisting community members apply for FEMA financial relief, and supporting community members at county townhall events. One program assistant shared: “we went door knocking, and because if the community [saw] us with FEMA, they were a little more apt to talk to them and say, okay, let me put my registration in.”

Describing the RADx-UP-funded project, a program manager expressed appreciation for the funding flexibility, which allowed the organization to address community-identified needs when offering COVID-19 testing services: “If they hadn’t been flexible, we wouldn’t have been able to develop our health worker program and also hire other staff members that have supported the program. In my experience with other contracts that we did have, a lot of them had a lot of limitations.”

### New investments may not translate to long-term sustainability

The interviews took place as RADx-UP and numerous other pandemic-oriented funding programs were expiring. The CBOs remained committed to continued health promotion initiatives. However, due to funding limitations, top-down intervention models, and project-based funding cycles, respondents felt that the developments made during the pandemic were unsustainable. CBOs struggled with how they would maintain the new staff and organizational capacity as pandemic funding decreased. During the time of data collection, all three CBOs were in the process of seeking new funding sources. It was unclear if their goals to continue their health promotion efforts would come to fruition. Because they were dependent on funding and partnerships, they would continue operating with limited resources, perpetuating the cycle of ‘doing more with less.’ Respondents described three key challenges that affected the sustainability of their health promotion initiatives.

First, CBOs reported that the inflexibility of many funders made it challenging to continue health promotion initiatives. Respondents felt that health programs were often not developed with specific communities in mind. In their experience, county and state organizations developed grants without input from smaller CBOs such as theirs. A program manager shared: “Instead of [the county] bringing us from the get-go to the table and say ‘Ok, what is it that you need? What is it that the community wants?’ they come from a point of ‘We know what you probably need.’…. That is such a waste of time and of impetus when *we* know from the beginning where the gaps are and what we lack.”

Second, the CBOs reported major challenges and limitations to funding CHW/*promotora* programs and providing fair payment to these essential staff. A program manager explained that many CHWs/*promotoras* “are not authorized to work in the United States. So, the default set up for agreement was to hire their services as independent contractors.” This approach allowed CBOs to pay them through stipends, and a director reported, “we are looking for grants that allow us to put stipends on it. But the stipend model is okay for emergencies, but in the long term it’s not the right model.” These challenges were reflected by the CHWs/*promotoras* themselves. One shared: “The truth is that this work is semi-voluntary. The time we put in doesn’t align with what we are paid because sometimes, the majority of the time, we come out negative in terms of pay.”

Third, grant reimbursement processes created financial burdens for CBOs, particularly those that were smaller and had fewer financial reserves. They reported that grants were often set up as fee-for-service where they would be reimbursed after specific deliverables were completed. It was challenging for CBOs to cover their costs as they started new programs that the grant would later reimburse. “It is unrealistic to get a contract of $250,000 for a year and expect us to have that kind of cash flow, to be able to front all of the money. And then deal with all of the invoicing, including all the receipts, all the reports for salaries, and then wait 90 days to get reimbursed. That whole system needs to change… Reimbursement grants are just a nightmare,” reported a director. Another, similarly, commented: “Organizations such as ours cannot start without some seed money.” This was particularly the case for federal, state, and county grants. One CBO reported that they were engaged in advocacy to try to change reimbursement practices: “County grants are also reimbursable grants and that’s one of our things on our agenda is to change… because it is not sustainable to live off the reimbursement grant.”

Because of these challenges, the CBOs emerged from the pandemic grappling with the same ongoing challenge of short-term funding and project-based services and activities. Because of restrictions, lack of flexibility, and the limits of reimbursement processes, each CBO faced different options for how to continue their work on health issues. Generations Rising was in the process of securing new funding to develop their “health department” and transition staff who had been brought on during the pandemic to lead new health promotion initiatives. In contrast, the leaders from the other two CBOs were struggling to retain staff and expressed less certainty about their futures due to fewer established options for financial resources; one program manager observed: “You can see also the disparities in resources. Generations Rising is a much larger, well-resourced community-based organization that is developing *promotoras* and creating a health department. We are very project-based. Jobs are going to go away, you know, when the grant ends in August. We may have some partial hours with the other initiatives that are happening. But we don’t have the job security or the opportunity really to develop a health department.”

Despite different prospects, all three CBOs had concerns about their sustainability within project-based funding cycles. A program manager from Generations Rising explained: “We understand that as a nonprofit, we are dependent on donations and these partnerships. But we need a consistent [funding] source that can be renewed and extended. We’re very willing to produce reports and narratives and everything just to see the work continue. It is nerve-wracking when you see that something is coming to an end.” Another noted, “This cycle of just constantly having to look for funding is very stressful.”

## Discussion

In this study, we examined the experiences of CBOs who served Latino communities to understand the factors that shaped how they adapted during the pandemic and how this work influenced their sustainability and priorities. We interviewed staff at multiple levels of three CBOs, providing perspectives from leadership and direct services staff. We identified three main themes that highlight both the strengths of Latino-serving CBOs and the challenges that they face: First, CBO staff who were bilingual, bicultural, and mission-driven were key resources in providing tailored interventions to Latino communities. Second, the pandemic led to an infusion of new funding that allowed CBOs to expand not only staff capacity but also organization capacity to engage in health promotion initiatives. Third, despite this burst of resources, there was evidence that the ongoing lack of flexibility from funders, challenges to providing pay to immigrant CHWs/*promotoras*, and the financial burdens of grants left all three CBOs in circumstances similar to those prior to the pandemic. We discuss these findings and provide recommendations to address ongoing challenges.

The findings highlight the critical role of bilingual, bicultural staff in supporting Latino-serving CBOs in meeting their missions. This is consistent with previous research that highlights the effectiveness of the CHW/*promotora* model [25]. CHWs/*promotoras* work at the intersections of community commitment and evidence-based health promotion, ensuring that interventions are delivered by individuals with deep community knowledge [8]. The CHW/*promotora* model is also recognized as an approach for building community leadership capacity [26]. As noted by one of our respondents, CBO leaders recognized the importance of empowering and engaging local residents to respond during an emergency. Despite being an established model and a feature of many Latino-focused CBOs, our findings also highlighted the ongoing challenges of funding CHW/*promotora* programs that provide fair payment.

Our study is unique in that we also identified the role of young Latino professionals in providing organizational capacity for culturally tailored programs in Latino communities. The funding made available during the pandemic created entry-level positions that brought new bilingual and bicultural professionals into health promotion. The role of new or young Latino professionals in Latino-serving organizations has received less attention. Our findings point to the need for future examination of recruitment, retention, and training of young Latino health professionals. There is evidence that health career pipeline programs for college students from underserved communities show promise; evidence suggests that participants have high rates of pursuing graduate training in health careers [27]. One program evaluation found that connections with institutions and organizations that had established relationships with young professionals were most effective for recruiting them into a pipeline program [28]. Latino-serving CBOs, for example, could be a vital source for connecting young professionals with health careers.

Our findings indicate that the infusion of resources to address the COVID-19 pandemic resulted in new organizational capacity to address health inequities. For at least one of the organizations in our study, the pandemic resulted in the expansion of their organizational mission to include health promotion and establish new programs that built from their new capacity. However, the pandemic also revealed funding and resource limitations that reduce CBOs’ future ability to continue health initiatives and their long-term sustainability. These findings align with previous research that highlights how nonprofit CBOs are reliant on a system of project-based and, primarily, government funding; [11, 14, 19, 29–32] further, despite the potential benefits of an influx in government funding, tensions emerge as this funding is often more “top-down” [29]. Previous research indicates that to maintain financial stability CBOs have to adapt to changing external priorities, such as those of funders, and “market” demands [11, 12, 32]. As federal, state, local, and philanthropic funding continues to change, the insights from this study highlight how organizations are able to adapt their priorities to meet community needs.

The challenges in funding that we observed may contribute to inequities in resources for Latino-serving CBOs. These inequities are likely to continue or be exacerbated as funding landscapes continue to evolve. Previous research suggests that CBOs who can obtain government grants are those with larger bureaucratic and professionalized funding systems [31]. Another study found that nonprofits that offered more services were more likely to secure government grants, but that securing government grants was negatively associated with their operating efficiency and long-term sustainability [30]. These dynamics suggest that CBOs’ financial stability is dependent on a focus on fundraising rather than an ability to be responsive to the immediate needs of the communities that they serve, disadvantaging CBOs focused on being responsive to community needs [32]. Finally, a major impediment to obtaining funding is the financial burden of covering costs while waiting for reimbursements from funders. Recent research has begun to document this results in inequities for smaller CBOs who are often deeply embedded in communities [33]. 

### Future directions and policy recommendations

This is one of the first studies, to our knowledge, to focus on the experiences of Latino-serving CBOs during the COVID-19 pandemic. A key strength of our approach was to include participants at each level of the organizations. One limitation, however, is that our case study approach focused on the experiences of a single initiative that received the same federal funding. Future research should examine CBOs with a range of funding and staffing models and who focused on different aspects of pandemic response, such as vaccination or housing. Another limitation is that our findings focused on a period when pandemic funding was coming to an end rather than taking a more longitudinal approach that could observe new dynamics after the funding ended. Currently, as communities brace for federal funding cuts, it is critical to identify how organizations continue to adapt and sustain themselves. Future research should continue to track the trajectories of Latino-serving CBOs.

Our findings point to recommendations for fostering CBOs’ health promotion capacity and increasing funding. First, government agencies who fund CBOs should collaborate with CBOs to understand health issues in Latino communities. Such collaborations could ensure that funding is responsive to local health needs. They could inform how to design initiatives that provide flexibility in deliverables so that CBOs can tailor and adapt to community needs as well as changing circumstances (e.g., new crises). Second, funders should structure funding levels and reimbursement processes with CBO input. This includes being attentive to CBOs fiscal needs, such as their ability to receive payment through reimbursements, and their administrative capacity, such as staffing to handle subcontracting procedures [33]. Finally, CBOs and funders should advance professional development initiatives for young professionals who are bilingual and bicultural or from underrepresented communities. Funding could support internships as well as transitions to graduate or professional school through scholarships or mentorship programs. CBOs are an ideal partner for such initiatives and investment in young professionals can support the long-term sustainability of these critical CBOs.

## Conclusion

Without significant changes to the “topdown” expectations of many funders and reimbursement systems, the new staff and organizational capacity gained by Latino-serving CBOs during the COVID-19 pandemic may disappear as CBOs are required to pivot into new areas of work to sustain funding. Ultimately, ongoing support and investment are needed to ensure that the gains made during the pandemic are sustained.

## Data Availability

The datasets used and/or analysed during the current study are available from the corresponding author on reasonable request.
